# Medications and pregnancy: The role of community pharmacists – A descriptive study

**DOI:** 10.1371/journal.pone.0195101

**Published:** 2018-05-09

**Authors:** Hoi Ying Leung, Bandana Saini, Helen E. Ritchie

**Affiliations:** 1 Faculty of Pharmacy, University of Sydney, Sydney, New South Wales, Australia; 2 Woolcock Institute of Medical Research, Sydney, New South Wales, Australia; 3 Discipline of Biomedical Science, Sydney Medical School, University of Sydney, Sydney, New South Wales, Australia; Karolinska Institutet, SWEDEN

## Abstract

**Background:**

Safe use of medications during pregnancy requires a comprehensive understanding of risk-benefit profiles for individual treatments. Pharmacists are supported in this aspect by clinical information agencies (e.g. MotherSafe, a telephone-based teratogen information service) and reference texts. To what extent and for what reasons Australian pharmacists utilise these services/resources are yet unknown. Further, debate on replacement of conventionally defined medication safety in pregnancy categories (A, B1-3, C, D, X) by narratively stated safety evidence may affect pharmacists’ routine practice. This study aimed to gauge pharmacists’ experiences and resource needs in undertaking support roles regarding gestational drug use.

**Methods:**

Semi-structured interviews (audio-recorded or documented using field notes) were performed with community pharmacists in Australia and transcribed verbatim. Inductive thematic analysis was conducted using the NVivo software (Version 11, QSR International).

**Results:**

Data saturation was achieved with 24 interviews. Qualitative data yielded 5 emergent themes: barriers to effective counselling, patient trust, risk perception, role definition and practice support needs. Overall, participants relied on pregnancy categories, were risk averse and cautious in offering advice. Currently available data for unclassified and category B therapeutic agents (limited human data) were deemed inadequate. Reluctance to use the proposed narrative system was also expressed.

**Discussion:**

This study highlights key barriers in the provision of maternal care by pharmacists and the potential tension present if the existing category system is replaced by a narrative one. These need to be addressed through training and development of practice support resources to enhance pharmacists’ skills in evidence-based risk estimation and communication.

## Introduction

In 2012, there were more than 200 million pregnant women worldwide [1]. Over the past 30 years, the number of women taking medications during their pregnancy has doubled, and it is now estimated that 90% of pregnant women consume at least one medication during gestation [2]. This could be attributed to pre-existing (chronic) or conditions that have developed as a result of the pregnancy. Some of these conditions inevitably require medications to address symptoms. Common disorders which warrant pharmacological intervention in pregnant women include asthma, depression, cardiovascular conditions, renal disease and diabetes. If left untreated these conditions could potentially have adverse effects on the fetus or require post-natal care of the new born [3].

As pharmacists, dispensing medications to this patient group requires cautious consideration due to potential deleterious effect of the medication on the mother and developing fetus. Factors such as embryonic/fetal age, drug dose, duration of use, risk-benefit profile, as well as changes in pharmacokinetics/pharmacodynamics parameters during pregnancy must be carefully considered [3]. Although most medications are non-teratogenic [4] and may be used safely in pregnancy, consumers often have concerns about the potential harmful impact on the unborn child. In the wake of the thalidomide saga, these concerns are sometimes inflated by media reports [5, 6]. Overestimation of teratogenic risks among consumers could result in non-adherence to needed treatment and sub-optimal control of maternal conditions, jeopardizing the health and safety of both mother and fetus [5]. Consequently, health professionals play a critical role in allaying patients’ concerns and disseminating evidence-based information.

Community pharmacists are key players in managing medication user during pregnancy as they are often the first line of contact and the last professional seen by patients after medicines have been prescribed. Equipped with knowledge of pharmacotherapy, as well as skills in health education and chronic disease management [7], pharmacists could help prevent drug-related issues by assessing the likelihood of fetal exposure and reviewing prescriptions to identify any dose errors [7], as well as potential drug interactions (including drug-food or drug-herb). Furthermore, given the specific expertise of pharmacists in terms of medication and their use, pharmacists are often consulted by other health professionals, and this would be the case for medication use in pregnancy also. As a result, their level of clinical confidence and knowledge regarding gestational use of medications are important variables for optimal outcomes in pregnant women. Research in Australia exploring the role of community pharmacists in optimising medication regimens for pregnant women is, however, lacking.

Australian pharmacists and other health professionals have clinical practice support offered through agencies such as MotherSafe and NPS MedicineWise. Mothersafe is a hospital-based teratogen information service in New South Wales (NSW) that aims to provide over-the-phone comprehensive advice for women and health practitioners, similar to Motherisk in Canada, MotherToBaby in the USA and Bumps in the United Kingdom (although the latter two provide resource material only). Drug information resources are also available in other Australian states, such as the Royal Women’s Hospital in Victoria [8]. Another clinical reference for pharmacists and other professionals in Australia is the Prescribing Medicines in Pregnancy database maintained by the Therapeutic Goods Administration (TGA). This database utilises the conventional classification of medications into non-hierarchical categories A, B1-3, C, D and X [9]. The categorisation provides a summary estimate of medication safety and allows professionals to quickly gauge the risk level associated with the use of any particular drug during pregnancy (Table 1) [Note: it excludes most topical agents, diagnostics, as well as complementary medicines, and is relevant only if medications are taken as recommended i.e. no overdose/off-label use]. The original intent of the risk classification system was to provide health practitioners the necessary information to ensure effective and safe use of medications when prescribing or counselling [10]. Nevertheless, there are mounting concerns about professionals simply associating an increasing level of risk with each letter in an alphabetical order (e.g. considering drugs in category B safer than those in category C), but not clearly distinguishing varying risks of fetal toxicity between drugs in the same category [2]. Drug allocation into a category is also based on the quality and amount of data available rather than the nature, severity or incidence of fetal risks [11]. Therefore, dependence on the classification system may lead to risk misinterpretation and ill-informed clinical decision-making [10]. For these and other reasons, the US Food and Drug Administration (FDA) abolished the category system in 2014. In 2015, the Australian Medicines Handbook (AMH) also removed pregnancy categories from their drug monographs in favour of narrative descriptions [12] (Table 2) (although the approved Product Information for all drugs in Australia still retains the letter risk categorisation system). The removal imposes greater responsibility on health professionals to ensure patient safety [2] with the need to comprehend evidence and make clinical judgements when prescribing/supplying medications to pregnant women.

**Table 1:** Australian categorisation system for registered medicines in pregnancy TGA [13].

Category	Definition
**A**	Drugs which have been taken by a large number of pregnant women and women of childbearing age without any proven increase in the frequency of malformations or other direct or indirect harmful effects on the fetus having been observed.
**B1**	Drugs which have been taken by only a limited number of pregnant women and women of childbearing age, without an increase in the frequency of malformation or other direct or indirect harmful effects on the human fetus having been observed. Studies in animals have not shown evidence of an increased occurrence of fetal damage.
**B2**	Drugs which have been taken by only a limited number of pregnant women and women of childbearing age, without an increase in the frequency of malformation or other direct or indirect harmful effects on the human fetus having been observed. Studies in animals are inadequate or may be lacking, but available data show no evidence of an increased occurrence of fetal damage.
**B3**	Drugs which have been taken by only a limited number of pregnant women and women of childbearing age, without an increase in the frequency of malformation or other direct or indirect harmful effects on the human fetus having been observed. Studies in animals have shown evidence of an increased occurrence of fetal damage, the significance of which is considered uncertain in humans.
**C**	Drugs which, owing to their pharmacological effects, have caused or may be suspected of causing, harmful effects on the human fetus or neonate without causing malformations. These effects may be reversible. Accompanying texts should be consulted for further details.
**D**	Drugs which have caused, are suspected to have caused or may be expected to cause, an increased incidence of human fetal malformations or irreversible damage. These drugs may also have adverse pharmacological effects. Accompanying texts should be consulted for further details.
**X**	Drugs which have such a high risk of causing permanent damage to the fetus that they should not be used in pregnancy or when there is a possibility of pregnancy.

**Table 2:** Current Australian categorisation system vs. FDA’s (proposed) narrative system for daclizumab (ZINBRYTA).

TGA-approved Product Information [14]	Narrative descriptions adapted from FDA [15]
**TGA (Australia)** **USE IN PREGNANCY (CATEGORY B3)** There are limited data on the use of ZINBRYTA in pregnant women. IgG antibodies are known to cross the placenta and placental transfer of daclizumab was observed in cynomolgus monkeys. In cynomolgus monkeys, administration of daclizumab at subcutaneous doses of up to 200 mg/kg/week during the period of organogenesis did not produce fetal malformations or variations, but fetal loss was increased at the high dose, associated with serum AUC exposure about 140 fold the AUC exposure expected in patients at the recommended clinical dose. The no-effect dose (50 mg/kg/week) was associated with AUC exposure 33 fold the expected clinical exposure. In cynomolgus monkeys, administration of daclizumab at a subcutaneous dose of 50 mg/kg/week during the last two-thirds of pregnancy had no adverse effects on fetal or postnatal development up to 6 months of age. This dose resulted in a serum AUC about 55 fold the AUC expected in patients at the recommended clinical dose. ZINBRYTA should be used during pregnancy only if the potential benefit justifies the potential risk to the fetus	**FDA (USA)** **RISK SUMMARY** There are no adequate data on the developmental risk associated with use of ZINBRYTA in pregnant women. Administration of ZINBRYTA to monkeys during gestation resulted in embryofetal death and reduced fetal growth at maternal exposures greater than 30 times that expected clinically. In the U.S. general population, the estimated background risk of major birth defects and miscarriage in clinically recognized pregnancies is 2–4% and 15–20%, respectively. The background risk of major birth defects and miscarriage for the indicated population is unknown. **DATA** (ANIMAL DATA) In monkeys administered ZINBRYTA (0, 10, 50, or 200 mg/kg) weekly by subcutaneous injection during organogenesis (gestation days 20 through 50), there was a decrease in fetal body weight and crown-rump length, and an increase in embryofetal death at the highest dose tested. Plasma exposure (AUC) at the no-effect dose of 50 mg/kg was approximately 30 times that in humans at the recommended human dose (RHD) of 150 mg. In monkeys administered ZINBRYTA (50 mg/kg) weekly by subcutaneous injection from gestation day 50 to birth, there were no effects on pre-or postnatal development for up to 6 months after birth. Plasma exposure (AUC) at the administered dose was 55 times that in humans at the RHD

Evidence suggests that medication counselling and appropriate risk communication could improve patient adherence to needed treatments and avert termination of wanted pregnancies [16, 17]. It is therefore crucial for pharmacists to possess the ability to confidently and appropriately provide relevant recommendations, as well as to alleviate the concerns of pregnant drug users. With the growing presence of well-informed (or misinformed) consumers and the ongoing debate about potentially removing conventional letter risk categories, pharmacists will need robust practice support. Thus, gaining an understanding of their current needs is pivotal to enable future resourcing.

### Aim

To gauge pharmacists’ practice experiences and resource needs in undertaking support roles regarding gestational drug use.

### Objective

To explore:

- Pharmacists’ attitudes and practice approaches in counselling pregnant women.
- Pharmacists’ opinions about the conventional risk classification system versus the proposed narrative system.

## Methods

Semi-structured interviews were conducted with Australian community pharmacists to attain insights into their experiences when providing medication advice for pregnant women.

### Ethics approval

The University of Sydney Human Research Ethics Committee (Protocol No. 2017/504) granted approval for this study protocol.

### Participant recruitment

The main strategy for recruitment included purposive convenience sampling; snowballing was then further used to enhance the sample size.

1. Purposive convenience sampling: Pharmacies in Western and South Western Sydney suburbs, identified via online Yellow Pages, were purposively selected as these areas (e.g. Blacktown, Auburn) have the highest birth rates in the state of New South Wales[18] and therefore relevant pharmacists were likely to possess significant experiences in counselling pregnant women. The approach to recruit participants within these areas was convenience based, i.e. pharmacists known to the research team to be working in the suburbs of interest were contacted using post, email or phone invitation.
2. Passive snowballing approach: All participants were requested, if they agreed, to forward information about this project to appropriate colleagues who met the inclusion criteria.

Sampling continued until a reasonable diversity in participant characteristics and data saturation were achieved.

Inclusion/exclusion criteria: Eligible participants had to be fully registered pharmacists with a work experience of at least 6 months. Interns or pharmacists registered for less than 6 months were excluded.
Consent: All eligible participants were provided with project information and requested for written consent to participate. The consent statement included willingness to allow interviews to be audio-taped. Prior to actual interview conduct, verbal consent was sought again to audio-record the interview.

### Interview design and data analysis

Interview conduct was facilitated using a semi-structured interview guide (S1 File), developed based on the clinical experience of the team and research literature. This included open-ended questions and prompts. Participants were also shown an example of Product information pertaining to drug use in pregnancy presented in the form of conventional alphabetical categories contrasted with a narrative style. Prompts were inbuilt into the guide to further a deeper discussion on the perspectives of pharmacists when counselling about gestational drug use. Interviews were performed by one interviewer (HYL) either face-to-face or over the phone with participants. Audio-recorded interviews were transcribed verbatim; in cases where participants preferred not to have the interview audio-taped, written notes were jotted down. In these cases, the notes were read immediately after the interview and written out in full to minimise recall bias.

Inductive thematic analysis was undertaken using NVivo software Version 11.0 (QSR International) for the data collected. Following interview transcription, initial codes were generated for the entire transcript set (S2 File). To enhance the veracity and objectivity of data interpretation, two researchers (one of whom was an experienced qualitative researcher) discussed and compared results following the first round of independent coding [19] for the initial 5 transcripts. Through ongoing comparison and refinement, a final coding structure was developed, which was then examined to explore patterns and relationships between codes and emergent ideas/concepts. Key ideas/concepts were discussed by the entire team with important themes finalised. In the last step, the ground researcher examined the themes by revisiting the coding structure to ensure that they represented codes in the entire data set [20].

## Results

### 1. Participant demographics

Just over half (24) of the 41 pharmacists approached consented to participate in the study (59% participation rate). Usual reasons expressed by invited pharmacists for non-participation were a lack of time or interest. Of the 24 interviews conducted, 18 (75.0%) were audio-taped while 6 (25.0%) were documented using field notes. No common characteristics were observed among participants who preferred not to have the interview audio-recorded. Interviewed pharmacists differed in their job type, practice environment (i.e. workload) and experience (range: ≤ 5–≥ 30 years). The majority of pharmacists graduated locally from Sydney, New South Wales (83.3%), the remainder graduating from Western Australia, Egypt, Pakistan and the UK (16.7%). Interviews took 20–25 minutes on average. Demographic details of participants are presented in Table 3.

**Table 3:** Demographic details of interviewed pharmacists (n = 24).

Characteristics	n (%)
**Age group**	
< 25 25–35 35–50 50–65 > 65	6 (25.0) 5 (20.8) 10 (41.7) 1 (4.2) 2 (8.3)
**Gender**	
Male Female	13 (54.2) 11 (45.8)
**Job type**	
General pharmacist employee Pharmacy owner Pharmacy manager Locum pharmacisT	15 (62.5) 4 (16.7) 1 (4.2) 4 (16.7)
**Pharmacy location**	
Shopping centre Medical centre Shopping strip Stand-alone	8 (33.3) 3 (12.5) 10 (41.7) 3 (12.5
**Pharmacy type**	
Banner/buying group* Franchise/Chain Independent	3 (12.5) 8 (33.3) 13 (54.2)
**Average number of pharmacists on duty per day**	
1 2 3 4	11 (45.8) 10 (41.7) 2 (8.3) 1 (4.2
**Number of non-pharmacist staff per day (range)**	1–11
**Average number of prescriptions dispensed per day**	
≤ 100 101–200 201–300 301–400 401–500 501–600	3 (12.5) 11 (45.8) 6 (25.0) 0 (0.0) 2 (8.3) 2 (8.3)

*A banner/buying group refers to a group of retail pharmacies involved in joint promotion and advertising/collective purchasing to maximise economies of scale and wholesaler bargaining power

### 2. Interview outcomes

Participants varied in their level of experience of patients who were planning to conceive (range: estimated 1–14 presentations per week) or pregnant (range: estimated 1–20 presentations per week). Enquiries from women who were planning to conceive generally concerned vitamins/supplements and preconception advice (e.g. pregnancy tests, methods to increase chances of conception). Whereas, questions from pregnant women were related to medication safety and the management of pregnancy-induced (e.g. reflux, gestational diabetes) or generic health conditions (e.g. cold and flu, infection). Generally, Over the Counter (OTC) medications were a more common reason why consumers sought information, compared to prescription products. The Australian Medicines Handbook (AMH) (39.6%), Monthly Index of Medical Specialities (MIMS) (27.1%) and MotherSafe (18.8%) were reported to be the common clinical resources utilised by study participants when addressing pregnancy-related queries. Five emergent topics were identified from thematic analysis of the interview transcripts: *barriers to effective counselling*, *patient trust*, *risk perception*, *role definition and practice support needs*.

#### Theme 1: Barriers to effective counselling

Patient Attitudes. Some interviewed pharmacists expressed that patients often had preconceived notions about what is safe to use during pregnancy. These were likely to be based on their personal experiences or readily accessible information from the Internet which, may be inaccurate and needs to be clarified.

“Sometimes it’s the patients themselves…they have these preconceived ideas about what they need and what they don’t need, and what’s important and what’s not. So sometimes they have information that’s not necessarily accurate, and you have to sort of go through.” (PH#1)

Time Constraints. Most participants acknowledged that community pharmacists are extremely busy, which limits their ability to devote sufficient time to counsel pregnant patients and conduct in-depth research when addressing queries.

“But with the majority of pharmacies, time is of essence, people are in a hurry all the time and you may not have sufficient time to research things.” (PH#22)

#### Theme 2: Patient trust

Participants shared the view that most consumers who approached pharmacists directly for advice were open to receiving information and recommendations about medication use in pregnancy; participants felt they were entrusted with giving accurate information. Some participants noted that cultural or religious differences, may result in patient reluctance to talk about their pregnancy/lifestyles or disclose sensitive information. It was also suggested that patients coming to the pharmacy after visiting their general practitioners (GPs) tend to place more reliance on physicians’ expertise, which can put pharmacists in an onerous position if intervention is required to ensure adequate care.

“They have the trust that whatever their doctors have prescribed for them is the best for them. So we don’t come in that picture as a pharmacist.” (PH#9)

“Because sometimes it’s related to authority and patients’ perceptions, patients may trust the doctor more in cases like that than the pharmacist.” (PH#14)

#### Theme 3: Risk perception

The majority of participants reported that pregnant women could be extremely anxious about taking medications, particularly those who have experienced difficulties in conceiving. Such concerns were difficult to allay at times. Consequently, gaining patient trust and confidence was considered to be an integral part of counselling. Nevertheless, participants acknowledged that they felt “paranoid”, “daunted” and sometimes “hesitant” to provide advice for this patient group, attributable to a fear of liability. Active risk assessment was generally avoided, and most interviewed pharmacists felt “confident”/“comfortable” recommending drugs in category A only. Some participants also noted that they are “cautious” and would always cross-check references (utilise at least 2 resources) to ensure the safety of prescribed medications.

“And sometimes it is the matter of paranoia as a pharmacist, because you know there’s liability involved.” (PH#1)

“I don’t gamble on anything. I use category A as my symbol.” (PH#22)

#### Theme 4: Role definition

Reassuring pregnant women that certain medications are safe to use during pregnancy, providing them with evidence-based or additional information, as well as assisting them in the selection of appropriate therapeutic agents, were deemed to be the central role of pharmacists.

“Because for pregnant ladies, they need support when using medications. Besides what the doctor and obstetrician say, we could also help reinforce their advice and tell patients that the medications are safe to use so they don’t have to worry.” (PH#14)

Inter-professional Collaboration. Most of those interviewed considered that they played a secondary role to GPs when handling prescription-related queries from pregnant drug users. The majority expressed the view that physicians have equivalent resources and their own experience in prescribing medications. GPs were also perceived by participants to be more knowledgeable with considerable expertise in the pregnancy field (i.e. managing chronic diseases), possibly due to their training and frequent exposures to relevant patients.

“At the moment, they [doctors] don’t rely on pharmacists, particularly the older ones. Because they’re used to their own system of evaluating things.” (PH#11)

Conversely, as highlighted by some participants, GPs were less familiar with over-the-counter (OTC) products often needing a secondary opinion from pharmacists regarding medication-specific issues (e.g. dosing, suitable alternatives). Some also noted that their contact with GPs was usually indirect via patients, where pharmacists served to reassure/reinforce GPs’ advice. Direct collaboration occurred only in complicated patient cases where medications from more restrictive pregnancy categories were warranted. In such instances pharmacists actively referred patients to GPs/obstetricians who were perceived to be the primary decision-makers.

“I definitely think it’s mainly trying to get a secondary health care professional to examine the risks and identify them.” (PH#6)

#### Theme 5: Practice support needs

Though participants mostly agreed that providing information to consumers and health information about medication use in pregnancy was a role for pharmacists, they generally also felt the need for intensive practice support to fulfil this role.

Information Sufficiency. Most participants shared the same view that: (1) there is inadequate information for the use/safety of OTC products in pregnancy (including complementary medicines and vitamins/minerals with no categories assigned); (2) currently available information is not specific enough and lacks details. Only a minority suggested that existing information is sufficient as medication choices in pregnancy are limited.

“But the AMH (Australian Medicines Handbook) sometimes is not as in-depth and it’s sort of quite…just short.” (PH#18)

“I feel like as far as prescription drugs [are concerned], it’s quite easy to find information; things which are over-the-counter or herbal, I find it very difficult.” (PH#20)

Information Format. Interviewed participants expressed mixed opinions regarding the potential removal of conventional letter risk categories and replacement with narrative descriptions,. The majority preferred keeping the categories or utilising a combination of categories with narrative descriptions (categories as secondary references/reinforcement) as pharmacists are well-adapted to the classification system. Statements regarding categories included: (1) categories serve as a “quick reference system” that helps simplify the decision-making process in a busy working environment; (2) it is easier for patients to understand that “there are different levels of safety with medications in pregnancy” and (3) it is simpler for pharmacists to communicate risks as categories are “clearly defined”. Some participants also suggested that categories are utilisable by consumers whereas narrative descriptions could be bewildering to patients as they may lack the knowledge to process information and make their own choices. Only a few participants preferred narrative descriptions only. These participants, who seldom referred to the risk classification system, had a positive attitude towards the introduction of narrative labelling as drug-specific information would provide more elaboration and explanation.

“I think it [narrative labelling] is a positive and would probably give more specific information. It’s not just a banner for a particular category, like all the B2 and B3 ones, it would be specific to the drug which is quite useful.” (PH#12)

Nevertheless, participants generally acknowledged that the classification system is “outdated” and the specific categories B1-3 are “ambiguous” (also confusing to patients). Some also noted that the given information is based on animal studies with limited relevance to humans and that categories are “black-and-white” while real-life clinical situations are not. Additionally, it was suggested that categories do not reflect the level of medication safety on a true scale and may be “misleading”. Most participants expressed concerns that: (1) having to read the information without categories provided would be more “time-consuming” and (2) there would be more ambiguity with a need to use one’s clinical judgement.

“So, if there is no category I feel like I would be a little bit murky, I wouldn’t be sure would this [medication] be a hundred percent safe, it would depend on how I interpret the data.” (PH#20)

Varying levels of confidence were reported by participants in relation to the use of narrative labelling. A minority felt insecure as they were uncertain about interpreting the given information as they were not trained in this aspect. A few participants noted that they may still require secondary references to be fully confident in providing recommendations.

“Yeah, I wouldn’t be confident to say, ok I’ve read this and I can give this out. I’ll probably, once again, double check somewhere else.” (PH#1)

Other participants reported that they would be more confident with time, as a better understanding of the pregnancy risks developed.

Information Quality. Most participants suggested that the quality of current resources could be improved by: (1) being more consistent, specific, clear and regularly reviewed; (2) introducing an evidence-based online pregnancy-specific database with inputs from experts in the field, containing risk summaries and information for herbal supplements. A minority recommended improving information accessibility by having an electronic alert (i.e. a software that immediately informs the pharmacist whether a prescription/OTC medication is safe or unsafe when scanned) or a category label attached to medication packaging.

“So, if you have the AMH (Australian Medicines Handbook) children’s dosing guide, you can always have a pregnancy one. Because it would have more inputs from gynecologists, those experienced in the field.” (PH#10)

“AMH and eMIMS (Monthly Index of Medical Specialties) only tell you about prescription [medications] but they don’t tell you about supplements or natural herbal things, so I feel like if there is a resource it would be helpful.” (PH#20)

Training and Incentives. Some of those interviewed suggested having education/training in the form of workshops or Continuing Professional Development (CPD) modules for medication use in pregnancy topics to keep pharmacists updated and get more understanding, although a few reported reluctance to participate due to time constraints.

“More workshops on how we can interpret this new narrative labelling, maybe do…because you know you have to do your CPD points every year, so probably do an activity on the transition from category labels to the narrative ones, how that’s going to affect us.” (PH#15)

## Discussion

This research aimed to explore the current practice and future support requirements of community pharmacists in assisting medication users who are pregnant or planning a pregnancy. The study also focused on pharmacists’ stance regarding the debate about using summarised alphabetical descriptors of medication safety in pregnancy versus a format where relevant data is presented in a narrative style. Given that community pharmacists are readily accessible primary health professionals, understanding these issues has importance for ensuring quality use of medicines by pregnant women. To the best of our knowledge, this is the first such study conducted in Australia. Findings suggested that pharmacists depended on summarised descriptors, assigned high levels of risk where data is uncertain and adopted a prudent approach in providing advice. They presumed a secondary role to physicians in decision-making for prescribed treatments in pregnant women. Participants felt unsupported in terms of the available information for non-prescription products and increasingly relied upon telephone support services in these instances. Clearly, these barriers need to be addressed and pharmacists should be upskilled, better supported and encouraged to play active roles in providing maternal care.

Results of our study illustrated that pharmacists appear to have considerable involvement in managing short-term pregnancy-induced ailments (e.g. nausea, reflux) by recommending over-the-counter (OTC) medications, rather than pre-existing (chronic) diseases which require prescriptions in which case consumers feel they had already received adequate information from their doctors. This is in line with previous survey data, suggesting that acute pregnancy-related conditions are often the main indication for gestational drug use [3, 21]. Interviewed pharmacists acknowledged their role in reassuring patients and reinforcing general practitioners’ (GPs) advice. Similar views have been expressed by pharmacists in the USA and Lithuania [22, 23], where participants reported a responsibility to assist in the selection of supplements/vitamins (OTC) for pregnancy and to reiterate physicians’ recommendations. Despite their willingness to deliver patient-centred care, pharmacists in our study described being confronted by several issues.

As highlighted in our results, it is obvious that the alphabetical risk categories (A, B1-3, C, D, X) have played a major role in participants’ decision-making for both OTC and prescription medicines. The tendency for health care providers and patients to overestimate teratogenic risks [17, 24–26] coupled with a fear of liability may hinder health professionals, such as physicians, pharmacists and nurses to actively explore relevant information [23, 24] (i.e. opt to avoid drugs or recommend ones in category A only). Together these may have contributed to a greater reliance on the risk classification system. Interviewed pharmacists were generally aware of the limitations of the categorisation system and were prepared to utilise additional resources. However most participants conceded that the categories form an immediate reference point that helps advise whether: (1) a medication is safe (i.e. category A) and could be dispensed and/or recommended; (2) there is a need for more reading (i.e. category B) or (3) it is essential to consult physicians/specialists or services like MotherSafe (i.e. categories B, C and D).

Participants misunderstood categories as hierarchical with medicines in an alphabetically higher order being mistakenly presumed as always safer. To avoid risk of liability, some of those interviewed delegated their responsibility in assisting medication selection to other professionals especially for drugs where use in pregnancy is recommended to be restricted. This may indicate a lack of confidence in advising pregnant women, as suggested in other studies where pharmacists reported a paucity of clinical experience and knowledge in this area [22, 27]. Several Australian studies have highlighted that other primary health professionals, such as GPs and practice nurses, also lacked clinical awareness in managing asthma and depression during pregnancy [28, 29], seeking information support in those cases [30]. Given the core role of pharmacists as highly accessible medication experts, it is necessary to develop their clinical skills to facilitate inter-professional collaboration in the provision of maternal care.

A scarcity of information for the safety of unclassified OTC products and the ambiguity associated with pregnancy category B were reported to be key barriers by participants. This represents a significant portion of prescriptions of medicines dispensed to women of child-bearing age (estimated to be 39% in Australia, [31]). Clinical hesitancy was expressed in providing concrete recommendations to pregnant drug users when medications lacked a pregnancy category (for example complementary medicines) or where human data was lacking or limited (category B). Notably, the inadequacy of scientific data for gestational use of herbal products was also reported by the study involving Lithuanian pharmacists [22]. This hesitancy is likely to be common given that the majority of prescription medications have not been evaluated adequately for safety of use in pregnancy [32]. In a recent survey of drug used by pregnant women and new mothers, 23% had no classification [33].

Clearly, pharmacists were aware that medicines exempted from the risk classification system are not always safe for use in pregnancy, as some could potentially interact with other medicines and produce unpredictable adverse reactions in the mother and fetus [13]. Given a consistent increase in gestational exposures to complementary medicines in Australia [34], there is evidently a need to provide pharmacists with more relevant information to better counsel patients in this aspect, as well as for category B drugs.

Pharmacists in our survey group predominantly relied upon The Australian Medicines Handbook (AMH) as the clinical resource when addressing pregnancy-related queries with a smaller number utilising the Monthly Index of Medical Specialities (MIMS). This is potentially problematic. Recent analyses of Product Information (PI) and Consumer Product Information of commonly prescribed drugs have highlighted the dissonance between pregnancy categorisation and accompanying information [35, 36]; for example,63% of PIs for drugs categories A (safe to use in pregnancy) were accompanied by text that contradicted that definition eg. doxylamine states “do not use during pregnancy”. This leads to situations highlighted in a recent survey of obstetric practitioners, where over 95% did not agree with Product Information recommendations regarding use in pregnancy [37].

As suggested earlier, the Australian Medicines Handbook (AMH) has made a decision to utilise narrative descriptions in drug monographs, with reasons similar to those of the US Food and Drug Administration’s (FDA) Our findings suggested that a potential extension of the removal to include Product Information for all drugs in Australia could have a significant impact on pharmacists’ clinical practice, for example, an over-estimation of risk [24].

This study identified two major concerns of pharmacists regarding the use of narrative labelling, mainly the ambiguity associated with data interpretation and data presentation/risk communication. These may be complicated by a lack of time in undertaking research and counselling as reported by participants. Even experienced health practitioners have difficulty evaluating experimental animal data in an organised and consistent manner, highlighting the need for supplementary resources to provide clear explanations for narratively stated information [38].

An incomplete understanding of the risk-benefit profiles of treatment by professionals could also impact patients who tend to assess risks subjectively depending on the wording in information texts [26]. Consequently, it is justifiable that training is imperative to develop pharmacists’ skills in data evaluation and risk articulation, so that crucial information can be communicated reliably to maximise patient health outcomes.

## Limitations

Our study involved some limitations. A biased sample of pharmacists was involved in the interviews, as only those who were interested, concerned and available chose to participate. In addition, interviewed participants were primarily from the same educational background and from a metropolitan populous area (i.e. trained and working in Sydney), which may limit the generalisability of our findings to the entire pharmacy profession. Nevertheless, generalisability is not the main focus of qualitative research and our study has offered an insight into the work and barriers confronted by pharmacists in supporting pregnant women, in particular the potential tension present with a shift from existing letter risk categories to narrative descriptions.

## Conclusion

Overall, our research findings highlighted key barriers in the provision of maternal care by community pharmacists, mainly the ambiguity associated with therapeutic agents which are unclassified or in category B, and with data interpretation using the narrative system. There is clearly a need to provide training options and modified pregnancy-specific clinical resources for pharmacists to improve their skills and professional knowledge in the maternity field. Future interventions should also enable them to take up a proactive role in counselling and be proficient in coping with unclear situations.

## Supporting information

S1 File Interview protocol. (DOCX)

S2 File Participant quotes by theme. (PDF)
