# Supplementary material for: Medications and pregnancy: The role of community pharmacists – A descriptive study
Source: PLoS One. 2018 May 9;13(5):e0195101. doi: 10.1371/journal.pone.0195101 (PMC5942805; doi:10.1371/journal.pone.0195101)
Supplement: S1 File — (DOCX) [file pone.0195101.s001.docx]

**Interview Protocol**

| **Questions** | **Prompts** |
| --- | --- |
| How often do women who are planning a pregnancy / pregnant present at pharmacy for advice/medications? | Many/Several/Few/No presentations a week |
| For what reasons do patients planning a pregnancy / who are pregnant usually present at pharmacies in your experience? | - Types of product queries (e.g. vitamins) - Generic issues - Specific pregnancy issues (e.g. maternal/infant sleep, treatment of pregnancy-related symptoms) - Specific health conditions ( e.g. chronic illness) - Common counselling points (e.g. vitamin supplementation) - General pharmacy services offered (e.g. baby check-up clinics) |
| Role of pharmacists in supporting pregnant women or women planning a pregnancy regarding medication use? | - Consultation - Medication Selection - Information Provision - Risk Communication |
| Key factors would you look for in suitable medications? | - Drug physicochemical characteristics - PI= Product category (A-X) - Mechanism of action of the medication - Patient health profile/specific issues e.g. allergy |
| What sources of clinical information would you use to check/confirm your decision? How satisfactory do you find these sources of information in real time? | - AMH - eMIMs - eTG - MotherSafe |
| How do you use pregnancy categories in your decision making? What do you believe were the advantages/disadvantages of this categorization system? |  |
| How do you think your practice will be affected by using the narrative system?  How confident do you feel about counselling a woman planning her pregnancy using the new narrative description rather than the categories? |  |
| How often are you consulted by other health care professionals about medications for use in pregnant women (or women planning a pregnancy)? | - If often: describe any recent communication (which medication/condition was it about, what was the query, and what advice was given) - If not very often: why this is the case |
| Key barriers you encounter in effectively counselling pregnant women or women planning a pregnancy about medication use? | - Training - Pharmacy setting/privacy issues - Patient trust/attitudes - Resources - Professional boundaries |
| Key resources you feel would help in effectively counselling pregnant women or women planning a pregnancy about medication use? | - Training (clinical and communication) - Resources - Practice incentives - 24/7 access to Mothersafe? |
