# Supplementary material for: Medications and pregnancy: The role of community pharmacists – A descriptive study
Source: PLoS One. 2018 May 9;13(5):e0195101. doi: 10.1371/journal.pone.0195101 (PMC5942805; doi:10.1371/journal.pone.0195101)
Supplement: S2 File — (PDF) [file pone.0195101.s002.pdf]

Theme 1 Quotes. Barriers to Effective Counselling

Subtheme 1. Patient attitudes

<Internals\\Transcript Ph#1.01> - § 1 reference coded [1.43% Coverage]

Reference 1 - 1.43% Coverage

Sometimes it's the patients themselves…they have these preconceived ideas about what they need and what they don't need, and what's important and what's not. So sometimes they have information that's not necessarily accurate, and you have to sort of go through

<Internals\\Transcript Ph#11.01> - § 1 reference coded [0.53% Coverage]

Reference 1 - 0.53% Coverage

if they do have any doubts, we will look up the references to try and clear them.

<Internals\\Transcript Ph#12.01> - § 3 references coded [4.92% Coverage]

Reference 1 - 1.66% Coverage

Often in these days patients are quite full of information themselves with access to the Internet and speaking to friends, things like that. But where they need assistance is more to make judgements and make sense of the information that they've been provided.

Reference 2 - 1.70% Coverage

I think most women are quite comfortable when you say something is category A, but as soon as you move further down, they're not really aware of the differences, and often to them, a category B is in their mind the same as a D. They automatically get quite hesitant.

Reference 3 - 1.57% Coverage

often it can be difficult for them to access the doctor as frequently as they would like because they've got so many questions and information being available on the Internet quite readily. So I think it's definitely an important part of our role

<Internals\\Transcript Ph#13.01> - § 1 reference coded [1.54% Coverage]

Reference 1 - 1.54% Coverage

A barrier would probably be when the woman is a bit nervous about taking medications in pregnancy because she is worried about the safety of her child

<Internals\\Transcript Ph#15.01> - § 1 reference coded [1.91% Coverage]

Reference 1 - 1.91% Coverage

Or the other one is because she already has children before, she thinks whatever she's tried is safe, despite new evidence coming out that suggests this isn't safe to use in pregnancy. It's up to her and I can't force her not to use it.

Subtheme 2. Time constraints

<Internals\\Transcript Ph#11.01> - § 2 references coded [2.12% Coverage]

Reference 1 - 1.11% Coverage

It's an issue of how much time you have to read these things as opposed to actually doing the work in pharmacy. Because I'm the only pharmacist, I have to do all the jobs

Reference 2 - 1.02% Coverage

the biggest problem is the economic pressure in pharmacy, everybody is, particularly retail…you try to maintain education as well as physically do the work.

<Internals\\Transcript Ph#12.01> - § 1 reference coded [1.30% Coverage]

Reference 1 - 1.30% Coverage

I think sometimes time can be an issue, especially if the information is not quite clear and you need to refer to someone like MotherSafe, you may be waiting in the line and have a few other things to do.

<Internals\\Transcript Ph#15.01> - § 1 reference coded [1.71% Coverage]

Reference 1 - 1.71% Coverage

I don’t have that much time because I am the only pharmacist on board, and there’s my assistant. So I can’t spend twenty minutes with patients going into details. I probably can spend a maximum of five minutes.

<Internals\\Transcript Ph#19.01> - § 1 reference coded [1.34% Coverage]

Reference 1 - 1.34% Coverage

- pharmacists may not always have sufficient time to counsel pregnant ladies

<Internals\\Transcript Ph#2.01> - § 1 reference coded [0.49% Coverage]

Reference 1 - 0.49% Coverage

I think no one is going to have time for training.

<Internals\\Transcript Ph#22.01> - § 2 references coded [2.12% Coverage]

Reference 1 - 0.88% Coverage

we don’t have the time to go into things quite as deeply as maybe is necessary to come up with an answer

Reference 2 - 1.24% Coverage

But with the majority of pharmacies, time is of essence, people are in a hurry all the time and you may not have sufficient time to research things

<Internals\\Transcript Ph#3.01> - § 2 references coded [1.66% Coverage]

Reference 1 - 1.49% Coverage

we are quite busy so if it’s over-the-counter, I’ll just give them like the safest option that I know of. If it is really still not ok, then I’ll just say: ‘maybe that’s the only option that you have’, just to persuade her this is the safest option.

Reference 2 - 0.17% Coverage

We just don’t have the time.

<Internals\\Transcript Ph#9.01> - § 2 references coded [1.06% Coverage]

Reference 1 - 0.15% Coverage

pharmacists are very busy

Reference 2 - 0.91% Coverage

Because pharmacists are really, really busy and I think the workload is not shrinking, it’s getting worse, worse in terms of it’s getting more and more

Theme 2. Patient trust

<Internals\\Transcript Ph#10.01> - § 2 references coded [1.48% Coverage]

Reference 1 - 1.11% Coverage

I think that when they (patients) actually ask you for information, it means that they want your advice, they want your input

Reference 2 - 0.37% Coverage

they trust you to give them the right ones

<Internals\\Transcript Ph#14.01> - § 2 references coded [1.50% Coverage]

Reference 1 - 1.34% Coverage

I think it's quite hard, that's why cases like that we would refer them to the doctor. Because sometimes it's related to authority and patients' perceptions, patients may trust the doctor more in cases like that than the pharmacist.

Reference 2 - 0.40% Coverage

different ethnic groups may have different perceptions of pharmacists

<Internals\\Transcript Ph#17.01> - § 1 reference coded [2.22% Coverage]

Reference 1 - 2.22% Coverage

- patients coming to the pharmacy would generally want and accept the pharmacist's advice

<Internals\\Transcript Ph#2.01> - § 1 reference coded [0.53% Coverage]

Reference 1 - 0.53% Coverage

And usually the patient would trust the doctor anyway.

<Internals\\Transcript Ph#20.01> - § 2 references coded [3.10% Coverage]

Reference 1 - 0.74% Coverage

but they want an answer from the pharmacist, usually they want me to give them an answer straight away

Reference 2 - 2.00% Coverage

Sometimes I'll tell them there's a free call phone number at the back of the pack, they can ring the company, just to check with the company because the company's got the data. But then people really want an answer from the pharmacist so in that situation it's quite difficult

<Internals\\Transcript Ph#21.01> - § 1 reference coded [4.01% Coverage]

Reference 1 - 4.01% Coverage

- Patients coming in to the pharmacy are quite open to pharmacists (e.g. willing to talk about their stage of pregnancy and even if they have previously lost a baby) because they want the best care

<Internals\\Transcript Ph#23.01> - § 2 references coded [3.18% Coverage]

Reference 1 - 1.84% Coverage

In some religions they don't feel comfortable talking about their pregnancy and/or maybe their living styles kind of thing. They're not confident talking about their pregnancy so some people are rigid enough, they say whatever the doctor has written, that's all they know

Reference 2 - 1.34% Coverage

most of our customers are more than happy when you say to them 'I'm going to talk to the doctor and confirm one thing, it's good for your safety, as well as mine.' Most of them they appreciate that

<Internals\\Transcript Ph#5.01> - § 2 references coded [1.76% Coverage]

Reference 1 - 1.26% Coverage

they don’t like to disclose sensitive information about themselves unless they have to. So that can be a bit of a barrier sometimes

Reference 2 - 0.50% Coverage

So it’s different if they are going to see a doctor.

<Internals\\Transcript Ph#8.01> - § 1 reference coded [2.12% Coverage]

Reference 1 - 2.12% Coverage

- Most customers are open and believe that pharmacists give them enough attention

<Internals\\Transcript Ph#9.01> - § 4 references coded [3.38% Coverage]

Reference 1 - 0.59% Coverage

patients believe that doctors are looking after them so they don’t need anyone else on top of that

Reference 2 - 0.87% Coverage

they have the trust that whatever their doctors have prescribed for them is the best for them. So we don’t come in that picture as a pharmacist.

Reference 3 - 0.70% Coverage

when they (patients) come in to get advice from us, they trust us and there is enough resources to actually look into

Reference 4 - 1.22% Coverage

But most of the pregnant women, when they come in and ask for advice from pharmacists, they have a trust in you and that’s why they come and ask you what to do while they’re pregnant, what dos and don’ts

Theme 3 Quotes. Risk Perception

<Internals\\Transcript Ph#1.01> - § 4 references coded [4.90% Coverage]

Reference 1 - 1.23% Coverage

the first thing I do, no matter how confident I am, I tell them ‘just give me a moment and I’ll have a look for you’. I’m not sure if I am the one who does this only, I just don’t feel comfortable unless I’ve seen the answer

Reference 2 - 1.27% Coverage

And sometimes it is the matter of paranoia as a pharmacist, because you know there’s liability involved. I want to make sure the patient is safe so I want to make sure that every recommendation I give is a hundred percent accurate.

Reference 3 - 1.32% Coverage

but of course sometimes as pharmacists, we can’t be more paranoid about the safety of patients and the child. We can leave mothers suffering with those minor complications that can be treated with relatively safe over-the-counter products.

Reference 4 - 1.08% Coverage

I understand what MotherSafe is trying to say, that I shouldn’t be too paranoid but once again, when you go into practice, it’s going to be difficult, if I don’t have that resources to support me.

<Internals\\Transcript Ph#12.01> - § 3 references coded [2.58% Coverage]

Reference 1 - 0.63% Coverage

Usually I would check at least two resources. I never really base information on just one resource.

Reference 2 - 1.41% Coverage

pregnancy is quite a significant time in someone's life and all health professionals often take quite a lot of responsibility when dealing with those patients, so in general, time needs to be assigned to these situations.

Reference 3 - 0.54% Coverage

It's always quite daunting when someone is pregnant and you're trying to give advice

<Internals\\Transcript Ph#13.01> - § 1 reference coded [0.71% Coverage]

Reference 1 - 0.71% Coverage

first of all, stick with the safest option and use that in pregnancy.

<Internals\\Transcript Ph#14.01> - § 3 references coded [1.22% Coverage]

Reference 1 - 0.47% Coverage

AMH is the basic one. We would also use other pregnancy information, just in case

Reference 2 - 0.45% Coverage

we would spend a lot of time with patients, especially when they are pregnant.

Reference 3 - 0.30% Coverage

because there's a lot of liability issues nowadays.

<Internals\\Transcript Ph#15.01> - § 1 reference coded [1.33% Coverage]

Reference 1 - 1.33% Coverage

So to always double check with females when they come in, whether she's pregnant or breastfeeding, because sometimes they are pregnant but they don't look pregnant.

<Internals\\Transcript Ph#17.01> - § 2 references coded [6.37% Coverage]

Reference 1 - 3.64% Coverage

- Finds it hard to counsel pregnant women, sometimes reluctant to provide them with advice because drug choices and information are quite limited

Reference 2 - 2.72% Coverage

- generally would recommend patients not to take medications; but if needed, may recommend ones in category A

<Internals\\Transcript Ph#18.01> - § 4 references coded [3.88% Coverage]

Reference 1 - 1.31% Coverage

obviously we would most commonly go for category A, because that’s the safest with a lot more research done and you feel confident recommending it as you know it’s definitely safe for the mother and baby to use

Reference 2 - 0.84% Coverage

I mean most of the time if it’s something not really upsetting, like a bit of hay fever, we try not to recommend anything in category B

Reference 3 - 0.91% Coverage

When it’s category B and there’s no human studies done and they are all animal-based studies, we don’t feel confident or comfortable recommending

Reference 4 - 0.81% Coverage

Yes it would take a bit more time but I’d rather be safe and spend that extra five minutes reading it (narrative description) and sort of understanding it

<Internals\\Transcript Ph#19.01> - § 3 references coded [6.34% Coverage]

Reference 1 - 2.58% Coverage

- It is always good to recommend patients something that you know is safe in pregnancy, rather than something we don’t have a wide experience of

Reference 2 - 0.67% Coverage

- Usually will use at least 2 resources

Reference 3 - 3.09% Coverage

- If it’s category B, then would become more cautious and would look through MIMS product information (animal studies) and other resources to make a holistic recommendation

<Internals\\Transcript Ph#21.01> - § 2 references coded [5.84% Coverage]

Reference 1 - 2.10% Coverage

- Always double check the safety of medications before supplying, even for ones prescribed by the doctor

Reference 2 - 3.75% Coverage

- Always cautious when recommending medications to pregnant women as wrong decisions by the pharmacist could result in lifelong damage for the baby and would carry a risk of being sued

<Internals\\Transcript Ph#22.01> - § 2 references coded [1.42% Coverage]

Reference 1 - 0.94% Coverage

it’s category A that I work on mainly because in my mind that’s the way to go so that everything comes out good

Reference 2 - 0.48% Coverage

I don’t gamble on anything. I use category A as my symbol

<Internals\\Transcript Ph#23.01> - § 6 references coded [5.60% Coverage]

Reference 1 - 0.27% Coverage

I usually don’t take risks, playing safe

Reference 2 - 1.37% Coverage

But if I need to make a rush in a situation, I’d always tell them at front that being on the safe side, I need some time for their sake, for their health, and most often they are more than happy to wait

Reference 3 - 0.91% Coverage

if (it’s an) over-the-counter medication and it’s in category B, I don’t take risks. I don’t suggest them, I always suggest the A ones

Reference 4 - 0.52% Coverage

No, always better to go and…

I: Like to double check?

P: Double check, yes.

Reference 5 - 0.35% Coverage

even if I have one percent doubt, I never take risks

Reference 6 - 2.16% Coverage

Sometimes in a busy pharmacy, if there is a long queue waiting and the customer next to you asks you questions about pregnancy, it doesn’t matter how long the queue is. Never take that risk. Give the customer a proper amount of time, check it, and never bluff on this situation because this is someone’s life and death

<Internals\\Transcript Ph#3.01> - § 1 reference coded [0.83% Coverage]

Reference 1 - 0.83% Coverage

I will say in the books, it’s not really recommended but it’s better for you to check with your doctor. That’s for my safe side to say that

<Internals\\Transcript Ph#5.01> - § 2 references coded [3.02% Coverage]

Reference 1 - 2.43% Coverage

I usually try to be more cautious if approaching a female person, when I’m recommending over-the-counter medications if they request something. Like I ask all the possible questions and make sure I’m recommending the right product for the right person

Reference 2 - 0.59% Coverage

But in general I try to stick to category A ones all the time

<Internals\\Transcript Ph#6.01> - § 3 references coded [1.69% Coverage]

Reference 1 - 0.41% Coverage

obviously trying to expand that research and look into other resources.

Reference 2 - 0.67% Coverage

I’d probably need to, maybe, accompany this with another database to feel very confident in making a recommendation.

Reference 3 - 0.61% Coverage

we just have to be confident in our knowledge base and if we’re not, try to develop that as best as we can

<Internals\\Transcript Ph#7.01> - § 2 references coded [2.85% Coverage]

Reference 1 - 1.61% Coverage

because anything from category A downwards I would tell them not to use it unless the doctor thinks the benefits are greater than the side effects

Reference 2 - 1.24% Coverage

you don’t want to be held responsible if there’s something wrong with the baby. You don’t want to take any risks.

<Internals\\Transcript Ph#8.01> - § 3 references coded [7.21% Coverage]

Reference 1 - 1.26% Coverage

- generally would just recommend category A drugs

Reference 2 - 2.28% Coverage

- Would consider non-pharmacological interventions whenever possible, just to play safe

Reference 3 - 3.67% Coverage

- if skeptical about product safety would refer to full PI pregnancy section, if nothing shows, would not recommend the product to patients

<Internals\\Transcript Ph#9.01> - § 2 references coded [2.25% Coverage]

Reference 1 - 1.64% Coverage

a lot of times they think they can’t use certain…for example, certain women they have chronic sinus issues and their inhalers like…they can be used (during pregnancy) and they’re (pharmacists) are under the impression that they can’t use anything and they (patients) suffer

Reference 2 - 0.60% Coverage

Anything from B downwards, I’ll call the doctor and let the doctor make the decision for the patient

Theme 4 Quotes, Role Definition

<Internals\\Transcript Ph#10.01> - § 1 reference coded [1.72% Coverage]

Reference 1 - 1.72% Coverage

sometimes when the prescriptions come along then I'll tell them what the idea behind it is or the rationale behind prescribing this. Just to keep them reassured as well what's the doctor doing.

<Internals\\Transcript Ph#12.01> - § 2 references coded [1.64% Coverage]

Reference 1 - 0.71% Coverage

other women are quite happy to take medications but just want the final reassurance that the medicine is safe.

Reference 2 - 0.94% Coverage

I personally find it quite rewarding as pregnant women are often less anxious when you allay their concerns or when you help them treat an ailment.

<Internals\\Transcript Ph#14.01> - § 5 references coded [5.54% Coverage]

Reference 1 - 1.14% Coverage

providing information is important, trying to explain the effects of medications and also telling them that it's ok to use…because a lot of pregnant ladies are very worried about using medications

Reference 2 - 0.59% Coverage

so it's a kind of reinforcement using documents to increase their confidence in taking the medication.

Reference 3 - 0.88% Coverage

They are worried about causing problems to the pregnancy, but the confidence we show them is important for them, to encourage them to use the medicine.

Reference 4 - 1.40% Coverage

Because for pregnant ladies, they need support when using medications. Besides what the doctor and obstetrician say, we could also help reinforce their advice and tell patients that the medications are safe to use so they don’t have to worry

Reference 5 - 1.54% Coverage

That’s why it’s important when we are working with pregnant ladies, to do a lot of research, like taking out the AMH and showing them, or taking out other information resources and sharing with them. This is important for them to gain confidence in using the drugs.

Subtheme 1. Inter-professional collaboration

<Internals\\Transcript Ph#10.01> - § 1 reference coded [0.68% Coverage]

Reference 1 - 0.68% Coverage

Because I guess they (doctors) have their own resources. They can look it up themselves

<Internals\\Transcript Ph#11.01> - § 2 references coded [3.48% Coverage]

Reference 1 - 2.47% Coverage

at the moment, they don’t rely on pharmacists, particularly the older ones. Because they’re used to their own system of evaluating things. The younger doctors may be more open to consultation about medication because it’s part of their training and they’re more familiar with that style of training; whereas the over 60s in particular, they’re used to making their own decisions.

Reference 2 - 1.01% Coverage

There’s always that issue of what we say to the patient about a treatment may be contradicted by the GP. And then all it does is that confusion to patients

<Internals\\Transcript Ph#12.01> - § 2 references coded [1.45% Coverage]

Reference 1 - 0.55% Coverage

I think they've (doctors) got access to the rest of the resources that we have access to as well

Reference 2 - 0.90% Coverage

But in the community, I think most GPs will have their own experience or will consult the obstetrician or gynecologist when making decisions.

<Internals\\Transcript Ph#13.01> - § 1 reference coded [1.00% Coverage]

Reference 1 - 1.00% Coverage

Maybe because they (doctors) are experienced, they probably see a lot of pregnant women on a regular basis.

<Internals\\Transcript Ph#14.01> - § 3 references coded [4.10% Coverage]

Reference 1 - 2.28% Coverage

But sometimes we also have to consider that it's a combined effort with other professionals. First of all, in complicated cases, we need to work with other professionals such as obstetricians and GPs, because they are the basic guides…they know the patient well. Then we work on the other side to make sure the medication is right for the patient, as well as the dosing frequency and strength.

Reference 2 - 0.42% Coverage

But category C ones would need a lot of other professional advice as well

Reference 3 - 1.40% Coverage

we have to weigh the risks and benefits. So in this case we're not the ones to decide…we have to talk to the specialist as to why the patient is using it, and the specialist needs to confirm with the GP and the GP…we'll be discussing together

<Internals\\Transcript Ph#18.01> - § 4 references coded [4.69% Coverage]

Reference 1 - 1.81% Coverage

If there isn’t any and I guess the condition is really upsetting the patient, then we try contacting the GP or having a chat with the GP and see whether they could recommend something or they might be happy with our recommendation and would follow up on the possibility of any side effects

Reference 2 - 1.44% Coverage

definitely no barriers in terms of getting into contact with the doctors and getting help, because we have a pretty good relationship with most of the doctors around the area. So any questions they’re usually quite happy to discuss

Reference 3 - 0.62% Coverage

It’s probably a bit tricky to get into contact with the specialists because they are always so busy

Reference 4 - 0.82% Coverage

So I guess it’s an over-the-counter thing, it’s not a prescription medication so I didn’t think he would have much details about it

<Internals\\Transcript Ph#19.01> - § 2 references coded [4.25% Coverage]

Reference 1 - 1.74% Coverage

- GPs would have already done some research before prescribing the medication to pregnant patients

Reference 2 - 2.51% Coverage

- There would only be more interactions when it comes to newly marketed medications like antiepileptic agents (limited clinical experiences)

<Internals\\Transcript Ph#2.01> - § 2 references coded [1.72% Coverage]

Reference 1 - 0.81% Coverage

Usually I think they check it themselves, like the doctor, before they prescribe it

Reference 2 - 0.91% Coverage

I think more like it is the professional boundaries. Usually the doctor has already decided…

<Internals\\Transcript Ph#20.01> - § 2 references coded [3.40% Coverage]

Reference 1 - 0.84% Coverage

I don't directly speak to other doctors about it but sometimes doctors indirectly referred customers to the pharmacy

Reference 2 - 2.56% Coverage

it's hard to bring up things to the doctor sometimes because in my experience, sometimes when you ring up the doctor and you say for example 'doctor, are you aware that this customer is on this medication and there's an interaction', a lot of doctors may say 'I already know', they are offended so it's hard to double check things with doctors sometimes

<Internals\\Transcript Ph#22.01> - § 8 references coded [10.07% Coverage]

Reference 1 - 0.72% Coverage

if it's too complicated I'd refer them to the doctor depending on what the problem is

Reference 2 - 1.07% Coverage

Not necessarily because if it's diabetes, they (patients) usually discuss that with their doctors and their doctors would be advising them

Reference 3 - 0.86% Coverage

I usually refer them (patients) to the doctor because most of our literature doesn’t go into that terribly deeply

Reference 4 - 1.01% Coverage

And the doctors, they have a deeper knowledge as to the teratogenic effects of different things in different categories

Reference 5 - 2.32% Coverage

all those down from category B and category C, doctors are specialized in that. Some of them have their own beliefs that some of those drugs are as safe as category A things, and they’ve studied that in their profession; whereas we don’t actually get to that type of studies

Reference 6 - 0.85% Coverage

I think they have their own set of resources and their own training and they make their own decisions

Reference 7 - 1.08% Coverage

So there is a grey area there…I’d prescribe things in A but she’s saying B and C would be just as good as A in certain instances

Reference 8 - 2.16% Coverage

This lady actually was a specialist in this sort of thing. She said that, because it was from her readings and she’s very knowledgeable, so as I’ve said there is an area of knowledge there that you need to try come across so that we know as much as they do

<Internals\\Transcript Ph#23.01> - § 3 references coded [1.80% Coverage]

Reference 1 - 0.31% Coverage

always consult the doctor. They know it better

Reference 2 - 0.94% Coverage

So interaction with doctors or other health care professionals is much more during weekdays rather than weekends. It depends on the timing

Reference 3 - 0.54% Coverage

They go on their references. They hardly ask a pharmacist before they prescribe.

<Internals\\Transcript Ph#3.01> - § 2 references coded [2.08% Coverage]

Reference 1 - 1.11% Coverage

I can say that maybe this one is better but at the end if it’s a prescription medication, we still have to refer back to the doctor. And the doctor may not agree with what we are saying.

Reference 2 - 0.97% Coverage

It’s really rare that a doctor will ring us asking whether it (a medication) is safe for use in pregnancy because they have the same information as eMIMS as well.

<Internals\\Transcript Ph#4.01> - § 2 references coded [8.76% Coverage]

Reference 1 - 4.90% Coverage

- Also, doctors have access to the same clinical resources as pharmacists

Reference 2 - 3.86% Coverage

- whether it is for pharmacists or doctors to make the final decision because ultimately, it is the doctor who prescribes the medication

<Internals\\Transcript Ph#5.01> - § 1 reference coded [0.45% Coverage]

Reference 1 - 0.45% Coverage

But most of the time, they (doctors) know or should know.

<Internals\\Transcript Ph#6.01> - § 2 references coded [2.65% Coverage]

Reference 1 - 0.82% Coverage

even to the point of communicating with specialists, doctors and speaking to IV clinics, seeing if we can order medicines for expecting mothers

Reference 2 - 1.82% Coverage

I definitely think it's mainly trying to get a secondary health care professional to examine the risks and identify them. Yes we do get questions about dosing, whether the medication is safe or not, but also more significantly we get information about whether it's accessible, whether there's a shortage of medication

<Internals\\Transcript Ph#7.01> - § 2 references coded [2.57% Coverage]

Reference 1 - 1.34% Coverage

I think doctors seem to think that for the prescribed stuff that they write, they probably have information to read about.

Reference 2 - 1.23% Coverage

they're always mainly focusing on prescribed stuff. Occasionally they'll call about some over-the-counter stuff.

<Internals\\Transcript Ph#8.01> - § 2 references coded [3.72% Coverage]

Reference 1 - 1.47% Coverage

- The majority of doctors seem not to rely on pharmacists

Reference 2 - 2.25% Coverage

- Being a doctor is prestigious so usually doctors wouldn't ask pharmacists for advice

<Internals\\Transcript Ph#9.01> - § 4 references coded [4.44% Coverage]

Reference 1 - 1.00% Coverage

I'm not saying that doctors don't know, obviously they know, but sometimes they will overlook because they are busy. So it's like a check and balance sort of a thing

Reference 2 - 1.13% Coverage

anything that's a prescription medication, anything from B downwards, we call the doctor and inform him that this is happening, having in mind that we could replace it with something safer

Reference 3 - 0.72% Coverage

I think doctors, when they're prescribing medications for pregnant women, they have already referred to their resources

Reference 4 - 1.60% Coverage

I believe when doctors and pharmacists are working in collaboration, basically GPs think that pharmacists are their resources, so they (GPs) ring them (pharmacists) rather than going through the books, and they ring them about what's the other thing they can give.

Theme 5 quotes. Practise support needs

Subtheme 1. Information sufficiency

<Internals\\Transcript Ph#1.01> - § 1 reference coded [0.79% Coverage]

Reference 1 - 0.79% Coverage

They don't have a lot of things for over-the-counter ones, prescription ones are probably more adequate but over-the-counter items, they won't.

<Internals\\Transcript Ph#10.01> - § 1 reference coded [0.47% Coverage]

Reference 1 - 0.47% Coverage

Obviously it's very general. It's not specific enough

<Internals\\Transcript Ph#12.01> - § 1 reference coded [0.91% Coverage]

Reference 1 - 0.91% Coverage

AMH can be quite good for some drugs, sometimes the limitation can be that it doesn't have a lot of information, it's not always very detailed

<Internals\\Transcript Ph#13.01> - § 1 reference coded [0.78% Coverage]

Reference 1 - 0.78% Coverage

but when it comes to multivitamins in pregnancy, it's difficult.

<Internals\\Transcript Ph#14.01> - § 1 reference coded [0.73% Coverage]

Reference 1 - 0.73% Coverage

basically the information we've got at the moment is not enough, is not clear, in relation to the safety of drugs in pregnancy

<Internals\\Transcript Ph#16.01> - § 1 reference coded [3.94% Coverage]

Reference 1 - 3.94% Coverage

- information available in AMH’s pregnancy section is quite limited without the categories (just says whether it’s safe or unsafe but descriptions are not detailed enough)

<Internals\\Transcript Ph#17.01> - § 1 reference coded [3.72% Coverage]

Reference 1 - 3.72% Coverage

- especially for categories other than A, the information often does not explicitly state whether a medication is safe or unsafe to use in pregnancy

<Internals\\Transcript Ph#18.01> - § 4 references coded [4.65% Coverage]

Reference 1 - 0.46% Coverage

but the AMH sometimes is not as in-depth and it’s sort of quite…just short

Reference 2 - 1.80% Coverage

A lot of information they give is very abbreviated, so they sort of just tell you this medication is category B and really that’s it. We know it’s a category B but maybe a bit more information on the risks involved and whether in different conditions it might be possible to recommend it.

Reference 3 - 0.94% Coverage

Most of the time we do find the information that we need to sort of help the patient, but sometimes there might be some questions that we can’t answer

Reference 4 - 1.44% Coverage

apparently she was recommended by her midwife to take raspberry leaf in early stages of pregnancy to ensure a smooth pregnancy, so that there wouldn’t be any issues in labor. I couldn’t find any information on taking raspberry leaf

<Internals\\Transcript Ph#19.01> - § 1 reference coded [2.62% Coverage]

Reference 1 - 2.62% Coverage

- there is a lack of information for OTC and herbal medicines, which often makes it difficult for pharmacists to reassure patients of their safety

<Internals\\Transcript Ph#20.01> - § 2 references coded [2.34% Coverage]

Reference 1 - 1.19% Coverage

I feel like as far as prescription drugs (are concerned), it's quite easy to find information; things which are over-the-counter or herbal, I find it very difficult

Reference 2 - 1.15% Coverage

It's hard when you get a supplement that has lots of ingredients in it, it's hard to research every single ingredient. I think that sort of database is lacking

<Internals\\Transcript Ph#21.01> - § 1 reference coded [4.32% Coverage]

Reference 1 - 4.32% Coverage

- there is a lack of information for complementary medicines; when patients really want to purchase a complementary product, pharmacist would contact relevant manufacturers such as Blackmores for more information

<Internals\\Transcript Ph#22.01> - § 2 references coded [2.22% Coverage]

Reference 1 - 0.76% Coverage

I don't think there needs to be anything more, maybe certain things could be more explicit

Reference 2 - 1.46% Coverage

I: Would you feel there is a lack of information for over-the-counter stuff or herbal medicines in the AMH?

P: Yes, it's something that the AMH doesn't do a great deal with

<Internals\\Transcript Ph#23.01> - § 2 references coded [2.14% Coverage]

Reference 1 - 1.71% Coverage

most of them you are not sure. Even if you go to their websites, sometimes it doesn't say whether it's safe (in pregnancy) or not. I mean even nutritional supplements, there are five to ten different nutrition in one and you can't check them one by one

Reference 2 - 0.43% Coverage

Prescription medicines are ok but not for over-the-counter ones

<Internals\\Transcript Ph#5.01> - § 1 reference coded [1.30% Coverage]

Reference 1 - 1.30% Coverage

the choice is not that great anyway for someone who's pregnant or breastfeeding, so the information that's available is already enough.

<Internals\\Transcript Ph#6.01> - § 1 reference coded [1.24% Coverage]

Reference 1 - 1.24% Coverage

And I guess the information that we can attain from resources like MotherSafe, which has a broader database accessing international research, would be amazing if we could filter that through to our current resources.

<Internals\\Transcript Ph#7.01> - § 2 references coded [1.12% Coverage]

Reference 1 - 0.71% Coverage

There are a lot of things that they don't have information about,

Reference 2 - 0.41% Coverage

but a lot of them there's no category

<Internals\\Transcript Ph#8.01> - § 2 references coded [5.04% Coverage]

Reference 1 - 2.30% Coverage

- herbal medicines are not categorized which may make decision-making difficult at times

Reference 2 - 2.73% Coverage

- AMH and eMIMS are not comprehensive enough in terms of information related to OTC and herbal medicines

Subtheme 2. Information format – use of pregnancy categories

<Internals\\Transcript Ph#1.01> - § 4 references coded [3.28% Coverage]

Reference 1 - 0.22% Coverage

but B it becomes a bit blurry sometimes.

Reference 2 - 0.92% Coverage

it's very ambiguous, because there's a couple of different Bs and some of them have been tested in animals and some haven't been tested. So it becomes a bit difficult.

Reference 3 - 1.32% Coverage

it's very variable between trimester to trimester…the safety of medications…and those categories don't distinguish that. So for example, something can be category A in this trimester and category D in another trimester, you'll never know.

Reference 4 - 0.83% Coverage

You can add both but I wouldn't say you have to remove the categories…I don't find them a bad thing, it's just a matter of they can do more with them.

<Internals\\Transcript Ph#10.01> - § 4 references coded [2.26% Coverage]

Reference 1 - 0.37% Coverage

I guess it's not very updated all the time

Reference 2 - 0.92% Coverage

It is definitely a quick reference like what I've learnt through university, I've learnt with categories

Reference 3 - 0.43% Coverage

The categories are almost like a short-cut thing

Reference 4 - 0.54% Coverage

it's sort of like you know what those categories mean already

<Internals\\Transcript Ph#11.01> - § 5 references coded [4.70% Coverage]

Reference 1 - 1.89% Coverage

the ambiguity lies in the statistics, because there are products which have been historically proven safe but because the evidence hasn't been documented, it will come out as a category C or B. People will sort of be less likely to recommend or use when in actual fact it may be safe to use

Reference 2 - 0.37% Coverage

I guess it depends on how often the medication's reviewed

Reference 3 - 0.23% Coverage

the information itself could be old

Reference 4 - 0.45% Coverage

I guess the categorization system is a quick way to red flag anything

Reference 5 - 1.76% Coverage

I would much prefer the categorization system, because it’s concise. Like if it is category A, then I can assume that it’s safe. If it’s anything else, then I would probably read through it to find out why it isn’t A. So in that way, it’s just a quicker reference system

<Internals\\Transcript Ph#12.01> - § 6 references coded [6.39% Coverage]

Reference 1 - 0.71% Coverage

I mean I will always check the product category and consider it, but it’s not the primary decision maker I guess

Reference 2 - 1.53% Coverage

they would be my first point of call because it’s quite a quick check and something could be excluded straight away, obviously if something is in category X or D, you would really refer the patient to MotherSafe if there’s no other options.

Reference 3 - 0.48% Coverage

I think A and X are the easiest because it’s usually quite clear ‘yes or no’

Reference 4 - 0.49% Coverage

It’s good that they’ve got a broad range of categories, especially with the B

Reference 5 - 1.71% Coverage

The disadvantage is that no clinical situations are exactly black and white, and often manufacturers would not update the categories due to financial constraints. So, the categories are not really updated as regularly as they should be, despite new evidence coming out.

Reference 6 - 1.46% Coverage

it provides everyone with a clear-cut…it's a decision-making tool where women are able to access these categories as well and they've got some understanding about there are different levels of safety with medications in pregnancy

<Internals\\Transcript Ph#14.01> - § 5 references coded [3.41% Coverage]

Reference 1 - 0.46% Coverage

but in other cases like B1 and B2, they are quite confusing to a lot of patients

Reference 2 - 0.43% Coverage

But the B1, B2 and B3 ones, they are very confusing, but still safe to use.

Reference 3 - 0.46% Coverage

It's simple to understand, usually it indicates that a medication is safe to use

Reference 4 - 1.61% Coverage

Sometimes it's easy for the patient to understand 'it's Category A, it would be safe' or 'it's Category B1, it's ok to use'. So sometimes it's just a symbolic idea, to put more confidence…because ultimately if a patient is really sick, we want the patient to use the medication

Reference 5 - 0.44% Coverage

the categories provide a layman understanding as to how safe a medication is

<Internals\\Transcript Ph#15.01> - § 3 references coded [3.65% Coverage]

Reference 1 - 1.42% Coverage

It’s more clear-cut. I can see as I am progressing from A to X, the severity of using the medication. It guides me in whether I’m going to sway away from the drug or towards it

Reference 2 - 1.17% Coverage

B, C and D are very unclear. You have to use your clinical judgement and to always look at the patient, it’s not always the same for every person

Reference 3 - 1.05% Coverage

If I see category A, honestly I would stop reading because I know that it’s safe, it’s been used in pregnancy and there’s no harm.

<Internals\\Transcript Ph#16.01> - § 4 references coded [6.88% Coverage]

Reference 1 - 2.01% Coverage

- Categories help reassure patients the extent of safety of a medication when used during pregnancy

Reference 2 - 0.88% Coverage

- categories sometimes can be misleading

Reference 3 - 0.60% Coverage

- categories serve as a quick reference

Reference 4 - 3.38% Coverage

- would prefer having the categories because it is easier for patients to understand the extent of safety of a medication when used during pregnancy

<Internals\\Transcript Ph#17.01> - § 2 references coded [2.57% Coverage]

Reference 1 - 1.55% Coverage

- categories are a quick guidance as to which drugs are safe to use in pregnancy

Reference 2 - 1.02% Coverage

- information (in categories) is often not specific enough

<Internals\\Transcript Ph#18.01> - § 3 references coded [1.61% Coverage]

Reference 1 - 0.30% Coverage

It (categories) gives you the peace of mind when recommending

Reference 2 - 0.83% Coverage

we see with a lot of things there are no human studies, they are all animal studies. So how relevant are we just being overly careful

Reference 3 - 0.34% Coverage

So personally I find the category B ones very ambiguous

<Internals\\Transcript Ph#19.01> - § 2 references coded [6.47% Coverage]

Reference 1 - 3.60% Coverage

- Categories are still necessary; if AMH says there is a lack of information for a specific medication, then the categories and animal data in MIMS would come into play (also should use own judgement)

Reference 2 - 2.87% Coverage

- act as secondary references; would give a quick idea as to category A means safe to use and category B means more reading is needed before making the decision

<Internals\\Transcript Ph#2.01> - § 1 reference coded [0.73% Coverage]

Reference 1 - 0.73% Coverage

Basically the whole category is based on animals, it’s not based on humans.

<Internals\\Transcript Ph#20.01> - § 3 references coded [4.78% Coverage]

Reference 1 - 1.47% Coverage

sometimes if I say it’s been used in a limited number of pregnant women, some women would want to know what that percentage is and it (category) doesn’t really say, so you know, it’s hard to explain that

Reference 2 - 1.81% Coverage

But with the categories, like what I’ve said I usually recommend category A, but if it’s category B, it’s still open for discussion. Yeah I find with the categories you can still discuss it with people and people sometimes still might want to use it

Reference 3 - 1.50% Coverage

I think I would prefer the existing one, because it’s so easy to look up something and then you see it is category A, straight away you feel confident, you can be like ‘ok it’s category A, I know it’s safe’.

<Internals\\Transcript Ph#21.01> - § 4 references coded [9.51% Coverage]

Reference 1 - 1.56% Coverage

- patients in general are familiar with category A but not with category B1-3

Reference 2 - 2.02% Coverage

- categories help guide pharmacists in explaining the potential risks associated with a medication to patients

Reference 3 - 2.47% Coverage

- sometimes (categories are) outdated and there could be grey areas; some medications may be used off-label but there were no ADRs reported

Reference 4 - 3.46% Coverage

- sometimes they may be misleading and contradicting (e.g. in MIMS, Polaramine is Category A but the description suggests that its safety in pregnancy is not established)

<Internals\\Transcript Ph#22.01> - § 4 references coded [3.44% Coverage]

Reference 1 - 1.12% Coverage

I: So you feel those categories could help you decide whether or not to recommend a medication to patients?

P: Most definitely, yes.

Reference 2 - 0.38% Coverage

I still think they should have the categories

Reference 3 - 0.45% Coverage

It's more clearly defined rather than having opinions

Reference 4 - 1.49% Coverage

It's quite a specialized thing and that's why I think the categories are the best because they help us make a decision and we're happy with our decision as it's clearly defined

<Internals\\Transcript Ph#23.01> - § 2 references coded [1.79% Coverage]

Reference 1 - 0.77% Coverage

The advantages are, it's a kind of thing which gives… it doesn't leave any grey area. It is either black or white

Reference 2 - 0.43% Coverage

previously we used to see the category straight away and answer

<Internals\\Transcript Ph#3.01> - § 5 references coded [6.01% Coverage]

Reference 1 - 1.29% Coverage

Most of the prescription medicines, if you check eMIMS, the pregnancy categories would be like B or C, which is a bit ambiguous, and it does not give a very good indication of whether the patient should use it or not

Reference 2 - 1.16% Coverage

the pregnancy category will be a good early recommendation for pregnant women but of course, it is not very like deep into information, because they will just say consult blah…information center.

Reference 3 - 1.10% Coverage

It is very clear; A is safe, anything after A is ambiguous, and then X is very unsafe. So it's a very quick way to notice whether a medication is safe or not, then give a recommendation

Reference 4 - 2.14% Coverage

we have been using this system for a long period of time so we are very used to it. The downside is, it doesn't go very deep into information, unless for some really old drugs, they have sufficient data; but for most of the new items like gliptins, you rarely have any data. So they will just give you a B or C, and then that means you don't know what to do.

Reference 5 - 0.32% Coverage

but B is really hard for me to give a recommendation.

<Internals\\Transcript Ph#4.01> - § 3 references coded [9.39% Coverage]

Reference 1 - 3.14% Coverage

- Less likely to consider product categories because they are not in true order in terms of the level of safety

Reference 2 - 3.66% Coverage

- If categorization system is on a true scale, it would provide a good indication in terms of how safe and unsafe a medication is

Reference 3 - 2.59% Coverage

- category B is ambiguous and there is a blurry line in terms of cut-offs between categories

<Internals\\Transcript Ph#5.01> - § 2 references coded [2.66% Coverage]

Reference 1 - 1.04% Coverage

I think it is clear enough in general, you know, it tells you which category is good or not good to be used

Reference 2 - 1.62% Coverage

there's a bit of confusion around the B1 and B2, because the recommendation says if the risk is greater than the benefit, you don't use it, so it's kind of a grey area

<Internals\\Transcript Ph#6.01> - § 5 references coded [4.76% Coverage]

Reference 1 - 0.24% Coverage

those categories were completely outdated

Reference 2 - 0.71% Coverage

I haven’t been utilizing the categorization system because I feel like that’s outdated. It’s not a great scheme to work upon

Reference 3 - 1.80% Coverage

doesn’t sound enough to make a recommendation upon. I guess that’s why we’re referring to AMH a bit more in a sense that they’ve changed their pregnancy recommendations and I feel that gives a bigger picture as to how the medicine works, rather than just relying on research that’s not fully examined and reviewed

Reference 4 - 1.11% Coverage

So the way that it was structured in terms of the categories B1, 2, 3 and then C, I felt weren’t reflective of how the medicines actually work and where they sit when it comes to pregnancy risk

Reference 5 - 0.90% Coverage

some people like the categorization system to know to pick from this and that selection, but when it comes to medications I think it’s more complex than that

<Internals\\Transcript Ph#7.01> - § 2 references coded [0.93% Coverage]

Reference 1 - 0.22% Coverage

we feel more assured (when using categories)

Reference 2 - 0.71% Coverage

More like when it’s not category A, there might be some ambiguity

<Internals\\Transcript Ph#8.01> - § 3 references coded [4.82% Coverage]

Reference 1 - 1.23% Coverage

- it is clear that categories C and D are unsafe

Reference 2 - 0.51% Coverage

- category B is vague

Reference 3 - 3.08% Coverage

- if a category is assigned to the drug, for example category B3, I would directly not recommend the drug to patients

<Internals\\Transcript Ph#9.01> - § 4 references coded [3.38% Coverage]

Reference 1 - 0.72% Coverage

Like A and X, you know, it's like a yes and no; but for B and C, they don't give you a clear answer so that's a pitfall

Reference 2 - 1.11% Coverage

quick reference and you're like 'ok, category A, I don't have to go into detail, off you go' so that sort of a thing. For the other categories, they're in the grey area, you don't know

Reference 3 - 0.73% Coverage

most of the time we already know, but we just need some sort of reinforcement, so that's why we just look at the category.

Reference 4 - 0.81% Coverage

to reassure that what we're saying is right. So that's why categories A, B, C and D is kind of a reinforcement to what we already know.

Subtheme 2. Information format – use of narrative descriptions

<Internals\\Transcript Ph#1.01> - § 1 reference coded [0.74% Coverage]

Reference 1 - 0.74% Coverage

Yeah I wouldn't be confident to say, ok I've read this and I can give this out. I'll probably, once again, double check somewhere else.

<Internals\\Transcript Ph#11.01> - § 3 references coded [4.55% Coverage]

Reference 1 - 1.65% Coverage

I think the biggest problem with not having a category is…you tend to read the information fairly quickly and when it says no adequate data, it doesn't let you know or doesn't indicate whether you should go for the side of safety or assume that it's safe

Reference 2 - 0.70% Coverage

I wouldn't feel confident, because I'm getting old and it's hard for me to keep up with all the medications.

Reference 3 - 1.39% Coverage

when you're going through university now, you're learning about the newer types of molecules coming through and they will become more the standard as they go forward. Whereas to me, I'm just not familiar with them.

<Internals\\Transcript Ph#12.01> - § 4 references coded [4.29% Coverage]

Reference 1 - 0.24% Coverage

I think I would see that as a positive

Reference 2 - 0.88% Coverage

Some people may find it's a time constraint because they need to read the information and spend a little bit more time making the decision

Reference 3 - 1.70% Coverage

However, given the fact that some of the categories are outdated and they don't give you all that much information, I think most pharmacists look to more than just the categories before making the decision, so in that respect I don't think it will be such a negative

Reference 4 - 1.47% Coverage

I think it's (narrative labelling) a positive and would probably give more specific information. It's not just a banner for a particular category, like all the B2 and B3 ones, it would be specific to the drug which is quite useful.

<Internals\\Transcript Ph#13.01> - § 3 references coded [1.90% Coverage]

Reference 1 - 0.35% Coverage

I think that would be much better.

Reference 2 - 0.93% Coverage

the website we use doesn't go according to categories. It's the same thing, it is narrative

Reference 3 - 0.62% Coverage

So we can use it as a back-up resource because it's helpful.

<Internals\\Transcript Ph#14.01> - § 7 references coded [6.63% Coverage]

Reference 1 - 0.31% Coverage

In fact, it would be very hard to explain to patients

Reference 2 - 1.61% Coverage

simple wording is much easier for patients to understand and perceive. Now, when you try to use the narrative labelling, there is a huge knowledge burden being put on patients' shoulders. Patients need to understand what all this concern is about and make their own judgements.

Reference 3 - 0.96% Coverage

a lot of patients, in terms of understanding, they don’t know what you are talking about. So it’s quite difficult for the laymen to understand the narrative labelling

Reference 4 - 0.59% Coverage

it’s good to give them (patients) a lot of information but at the same time, it can be very confusing.

Reference 5 - 1.04% Coverage

During pregnancy, they (patients) are very concerned about taking medications, and adding this (narrative labelling) into it, it would make them less likely to take the medication.

Reference 6 - 1.08% Coverage

but for pregnant ladies, they want to try to get rid of even the ten percent risk. So there is a lot of consideration or pressure being put on patients when using the narrative labelling

Reference 7 - 0.78% Coverage

If you give patients a lot of information related to side effects, how many percentage of this and that, that would only confuse them.

<Internals\\Transcript Ph#15.01> - § 7 references coded [8.17% Coverage]

Reference 1 - 1.37% Coverage

It’s not black-and-white, like before there’s category X and category A. But now it’s like ‘consider this safe to use in that’, we have to use all our clinical judgement

Reference 2 - 0.79% Coverage

I: So you think there are more uncertainties with the current ones?

P: Yes I feel like there are.

Reference 3 - 0.95% Coverage

It’s very similar to the category, just minus the category. Like what I’m reading is just re-worded with more numbers

Reference 4 - 0.36% Coverage

I think I have to use my clinical judgement.

Reference 5 - 1.33% Coverage

if there are no categories, I would have to read the whole thing and then use my own clinical knowledge. That’s the only thing. It’s going to be more time-consuming

Reference 6 - 1.12% Coverage

I: So would you feel more confident or less confident?

P: I think I’d feel less, because I was trained with A, B, C, D and X in university

Reference 7 - 2.26% Coverage

we’re not trained how to interpret this…something I have to do by myself. If I was at university and the categories were removed, I would have been guided but this (narrative labelling) is implemented after. So it’s extra work for me to do. I have to learn how to interpret this.

<Internals\\Transcript Ph#16.01> - § 2 references coded [1.83% Coverage]

Reference 1 - 1.39% Coverage

- practice would be made more difficult without the categories

Reference 2 - 0.44% Coverage

- more time-consuming

<Internals\\Transcript Ph#17.01> - § 3 references coded [14.34% Coverage]

Reference 1 - 6.77% Coverage

- if the labelling would explicitly state whether a medication is safe or unsafe to use in pregnancy then that would be useful (specific statements e.g. safe in 1st trimester, unsafe in 2nd and 3rd trimester) as it saves time and helps pharmacists make recommendations

Reference 2 - 3.44% Coverage

- if the labelling just says no adequate data then wouldn't find it that useful; such form of labelling would also be more time-consuming

Reference 3 - 4.13% Coverage

- having more information provided without explicitly saying whether it's safe or unsafe to use a medication in pregnancy doesn't really help with making judgements

<Internals\\Transcript Ph#18.01> - § 3 references coded [2.53% Coverage]

Reference 1 - 0.65% Coverage

I guess if it's got all the information written, then it might be just as good as the category labelling

Reference 2 - 0.34% Coverage

I think it would help, personally I find it would help

Reference 3 - 1.53% Coverage

I think knowing the risks, for me, would be better. I feel more confident knowing what I'm actually telling the customers and obviously by reading that, I could summarize it in an easy understandable way to the patient of what risks are involved

<Internals\\Transcript Ph#19.01> - § 2 references coded [4.27% Coverage]

Reference 1 - 3.09% Coverage

- not much of a problem because AMH has a similar form of labelling; it could be a bit of a read but it helps improve one's understanding of the drug when used in pregnancy

Reference 2 - 1.18% Coverage

- would still feel confident but would prefer having the categories

<Internals\\Transcript Ph#20.01> - § 3 references coded [4.49% Coverage]

Reference 1 - 1.02% Coverage

if you look up something and it just says safe to use or not safe to use, there would be no discussion and it would be just black-and-white

Reference 2 - 1.15% Coverage

I think it makes things a little bit harder, because the own (responsibility) is on the pharmacist…you're making the decision, like how you interpret the data

Reference 3 - 1.33% Coverage

So if there is no category I feel like I would be a little bit murky, I wouldn't be sure would this (medication) be a hundred percent safe, it would depend on how I interpret the data

<Internals\\Transcript Ph#21.01> - § 2 references coded [6.38% Coverage]

Reference 1 - 4.86% Coverage

- practice not really affected because it is just a rephrasing of information; used to always read information available and try to understand why a medication is placed under a certain category, so not basing judgement on categories only

Reference 2 - 1.52% Coverage

- would still feel confident but just need more time to read the information

<Internals\\Transcript Ph#22.01> - § 1 reference coded [2.16% Coverage]

Reference 1 - 2.16% Coverage

when we are asked questions and we have to make a decision and patient's health is premium, I'd prefer to have something that says 'yes or no' rather than trying to make up something in my own mind, when I may have other things in mind that are pressing me

<Internals\\Transcript Ph#23.01> - § 5 references coded [6.04% Coverage]

Reference 1 - 1.25% Coverage

Personally I would find it hard in the beginning. Maybe most of the pharmacists, they would consult their older versions of AMH in that case until they are familiar with the new style

Reference 2 - 0.65% Coverage

in the future this one would be much better than those categories, once I'm good enough in this.

Reference 3 - 2.74% Coverage

Now we have to read each and everything from the beginning, we'll do it a couple of times, and then it (the information) would stay somewhere in your mind. So that's why I'm saying in the beginning, yes it would be hard, but if it is being practiced again and again, this one is better because it elaborates much better rather than simplifying words as B3 that kind of thing. It's explaining everything

Reference 4 - 0.71% Coverage

I don't know in the beginning, I'm not confident at all. But my confidence would build up later with time

Reference 5 - 0.69% Coverage

As you’ve mentioned that AMH has taken off the categories, it’s going to make life harder to be honest

<Internals\\Transcript Ph#3.01> - § 5 references coded [6.17% Coverage]

Reference 1 - 0.30% Coverage

It would just take more time to assess information.

Reference 2 - 1.93% Coverage

Because if you just tell me this kind of study, it is also kind of limited data, unless you give me like a Cochrane Review. Otherwise, I would say there’s limited data and that means I have no point to look at it as well. That means I’ll jump back to the conclusion of saying limited data and I don’t know what to recommend.

Reference 3 - 1.90% Coverage

I think this part of the information is still important in terms of deep counselling or doing some kind of medication review or some kind of medication checks. Yes this would be very informative but in terms of just over-the-counter, normal counselling or recommendation, there’s too much to read in a busy environment

Reference 4 - 1.29% Coverage

in terms of time, the new one does not give me a good indication to say what immediately, so I have to go deep into the paragraphs and it will take me more time. But eventually we may end up with the same conclusion.

Reference 5 - 0.75% Coverage

just that if you have limited time and you have to read the whole paragraph, it’s not really practical in a community setting

<Internals\\Transcript Ph#4.01> - § 1 reference coded [9.07% Coverage]

Reference 1 - 9.07% Coverage

- would not like narrative labelling only because if the drug category is unknown, other relevant information would easily slip my mind. If, for example, I know the category is B, then I would immediately know that I need to read more to make the decision. Having narrative labelling only would be more time-consuming

<Internals\\Transcript Ph#5.01> - § 3 references coded [4.33% Coverage]

Reference 1 - 1.25% Coverage

I think that would be a little bit more confusing until I get used to it. Because at the moment, we used to look at the categories

Reference 2 - 1.40% Coverage

if you have to read about twenty lines before you decide if this medication is safe or not, I think it would be more confusing and time-consuming

Reference 3 - 1.68% Coverage

I think I would feel more confident. Because I have to read the information, which would give me the exact idea about what sort of studies were done and what were the results

<Internals\\Transcript Ph#6.01> - § 1 reference coded [1.39% Coverage]

Reference 1 - 1.39% Coverage

I like the fact that it’s actually in a narrative setting, purely because I can interpret and understand where the lack of information is, rather than trying to follow a categorization system and be misled by the numbering of the categories.

<Internals\\Transcript Ph#7.01> - § 2 references coded [3.94% Coverage]

Reference 1 - 2.02% Coverage

It depends on the way they word it I suppose but consumers do want some sort of like a ‘yes or no’ answer. If you start telling them grey stuff, then they think you don’t know too much

Reference 2 - 1.91% Coverage

if they are not going to put the categories I think there’s going to be a lot of grey things so you basically tell them that ‘it’s your choice, you take it at your own risk’.

<Internals\\Transcript Ph#8.01> - § 1 reference coded [5.12% Coverage]

Reference 1 - 5.12% Coverage

- would give me more courage (more confident) to advise with some medications because the need to read through all information when making the decision increases my understanding about the drug

<Internals\\Transcript Ph#9.01> - § 2 references coded [1.25% Coverage]

Reference 1 - 0.96% Coverage

it (narrative labelling) is good, it’s a bit of more work. We have to adjust our settings to it, because we have our mindsets based according to the categories

Reference 2 - 0.29% Coverage

The only pitfall is that it’ll consume more time

Subtheme 3. Information quality

<Internals\\Transcript Ph#1.01> - § 3 references coded [3.32% Coverage]

Reference 1 - 1.41% Coverage

So if I have like summaries, for example, if I have PPIs, proton pump inhibitors, and I have a summary of the sort of times they were given to pregnant women and then a recommendation at the bottom: safe to give within this trimester…that would be amazing.

Reference 2 - 1.56% Coverage

so they can go over like those different complications and different medications that you can or cannot give, and then give out some factsheets, something like that. That would be really good. But once again, time constraints, people are working, it's difficult to get people involved

Reference 3 - 0.35% Coverage

I need evidence-based resources to support any suggestion I make

<Internals\\Transcript Ph#10.01> - § 1 reference coded [1.51% Coverage]

Reference 1 - 1.51% Coverage

So if you have the AMH children's dosing guide, you can always have a pregnancy one. Because it would have more inputs from gynecologists, those experienced in the field.

<Internals\\Transcript Ph#11.01> - § 3 references coded [3.12% Coverage]

Reference 1 - 1.28% Coverage

It would be interesting to put the category at the bottom, rather than at the top. So in that way you would basically read it and get to the bottom, then maybe you would associate with the category

Reference 2 - 0.57% Coverage

it needs to be reviewed to keep it up-to-date. And I guess it has to be evidence-based.

Reference 3 - 0.53% Coverage

The important thing is to remind everybody about what's new and what's happening.

<Internals\\Transcript Ph#12.01> - § 3 references coded [2.43% Coverage]

Reference 1 - 0.92% Coverage

I think what would be more useful is to have more frequent updates on the categories or the recommendations in pregnancy in our regular resources

Reference 2 - 1.20% Coverage

that would be quite handy if there is a specific database for pregnancy with detailed information as to what you've suggested, the risk summaries, that would be helpful if they are online.

Reference 3 - 0.31% Coverage

it's something that we do need more resources on

<Internals\\Transcript Ph#14.01> - § 4 references coded [4.40% Coverage]

Reference 1 - 0.58% Coverage

But the thing is, if there is a book version, like the TG, for pregnancy, that would be even better.

Reference 2 - 2.14% Coverage

the TGA has to make an effort to ensure manufacturers…for example, the guidelines from manufacturers have to be very clear, is the medication safe to use…because when you look at a simple product like Claratyne, it doesn't really say whether it's safe to use in pregnancy but tells you to ask your doctor or pharmacist. That can be confusing to patients and pharmacists

Reference 3 - 0.65% Coverage

It's more to do with the inconsistencies (between resources) which is my concern, which is confusing to patients

Reference 4 - 1.03% Coverage

No one can really decide whether it's ok to use except the doctor. For us as pharmacists, we're not trying to avoid the responsibility, but why is there all that inconsistencies?

<Internals\\Transcript Ph#15.01> - § 2 references coded [2.98% Coverage]

Reference 1 - 0.60% Coverage

I'd like them to be more definite, to guide us more into what we should do

Reference 2 - 0.88% Coverage

I think they could have done both, they can still keep the categories and give us more information in details

<Internals\\Transcript Ph#16.01> - § 1 reference coded [4.05% Coverage]

Reference 1 - 4.05% Coverage

- Pregnancy information in AMH should be clear and more detailed e.g. having a pregnancy handbook embedded within instead of needing a separate resource to refer to (all-in-one)

<Internals\\Transcript Ph#17.01> - § 1 reference coded [7.49% Coverage]

Reference 1 - 7.49% Coverage

- Pregnancy information in current resources should be more specific, concise, with accurate data (e.g. should say when could a medication be given, is it safe or unsafe, would it do any harm to the unborn child) and without grey areas (should not say 'may or may not' but should say 'yes or no')

<Internals\\Transcript Ph#18.01> - § 3 references coded [4.11% Coverage]

Reference 1 - 2.79% Coverage

I've seen doctors and even gynecologists recommending things that are in category B, B2. And you know, double check with them and they might say from their experiences and monitoring of patients, they find that the medication works and there is no ill effect,

so coming down to the benefits and risks, they'll say it's worth going ahead with it. So it would be good if we have that sort of information, a bit more data to be able to help patients

Reference 2 - 0.43% Coverage

maybe a bit more information on pregnancy and medicines in pregnancy

Reference 3 - 0.24% Coverage

A bit more information for supplements

<Internals\\Transcript Ph#19.01> - § 1 reference coded [1.96% Coverage]

Reference 1 - 1.96% Coverage

- An online version of pregnancy guidelines which every pharmacy can have access to (similar to AMH and eMIMS)

<Internals\\Transcript Ph#2.01> - § 1 reference coded [0.43% Coverage]

Reference 1 - 0.43% Coverage

And also more details for risks and benefits

<Internals\\Transcript Ph#20.01> - § 4 references coded [3.88% Coverage]

Reference 1 - 1.46% Coverage

if there are some comprehensive resources which pharmacists could look up information for pregnancy, and if not just for pregnancy, like it's very difficult to find information for breastfeeding as well

Reference 2 - 0.84% Coverage

I want there to be some sort of incentives for pharmacists, because we are so busy already with so many other things

Reference 3 - 0.77% Coverage

I get a lot of questions about over-the-counter things, and I wish there are more resources for pharmacies

Reference 4 - 0.46% Coverage

more incentives, like some sort of remuneration or higher wages

<Internals\\Transcript Ph#22.01> - § 3 references coded [4.23% Coverage]

Reference 1 - 0.35% Coverage

There could be a herbal medicines handbook

Reference 2 - 0.60% Coverage

I think a medicines handbook for pregnancy would be a pretty good thing

Reference 3 - 2.16% Coverage

This lady actually was a specialist in this sort of thing. She said that, because it was from her readings and she's very knowledgeable, so as I've said there is an area of knowledge there that you need to try come across so that we know as much as they do

<Internals\\Transcript Ph#23.01> - § 1 reference coded [1.96% Coverage]

Reference 1 - 1.96% Coverage

if something is backing up, like a better software, we simply put the medication name on one side and on the other side it would say whether it's safe or not, for both prescription and over-the-counter…when I say over-the-counter it includes everything. This is going to be so much easier

<Internals\\Transcript Ph#6.01> - § 2 references coded [2.50% Coverage]

Reference 1 - 1.17% Coverage

Whether or not we can use a new categorization system that's more adequate and has a greater structure, with this narrative labelling, I think that might improve it and make it easier for some pharmacists

Reference 2 - 0.83% Coverage

the database could actually quickly go through current information about what's considered safe or not. That would help just to add extra support

<Internals\\Transcript Ph#7.01> - § 2 references coded [3.69% Coverage]

Reference 1 - 1.88% Coverage

I think it's about doing quicker like if they have a small label that you could put next to the product, like for any safe ones or the commonly used medicines in pregnancy

Reference 2 - 1.81% Coverage

whatever it's gaviscon, it's category A…then at least when we see it so many times, it sticks in our head, otherwise we spend all the time going back to check things

<Internals\\Transcript Ph#9.01> - § 1 reference coded [1.19% Coverage]

Reference 1 - 1.19% Coverage

if you want to develop a resource, develop it for the patients themselves, so when people come to us, we'll be able to give them the website or some sort of resources which they can always refer to

Subtheme 4. Training and incentives

<Internals\\Transcript Ph#11.01> - § 1 reference coded [0.22% Coverage]

Reference 1 - 0.22% Coverage

But it’s important that the opportunity for continued education is there and I think people will take advantage of it if it occurs

<Internals\\Transcript Ph#15.01> - § 2 references coded [3.07% Coverage]

Reference 1 - 2.07% Coverage

more workshops on how we can interpret this new narrative labelling, maybe do…because you know you have to do your CPD points every year, so probably do an activity on the transition from category labels to the narrative ones, how that’s going to affect us

Reference 2 - 1.43% Coverage

by giving us some questions and tests so that we can interpret it right. I think that would be helpful because we’re out of university so any training would be CPD or workshops.

<Internals\\Transcript Ph#18.01> - § 1 reference coded [1.66% Coverage]

Reference 1 - 1.66% Coverage

I do remember most of the CPD do touch on a bit of pregnancy, whether it’s contraindicated, just a small paragraph of it, like conditions and pregnancy type thing. But yeah a bit more specific would be good, like a training session just on pregnancy and medications

<Internals\\Transcript Ph#22.01> - § 1 reference coded [2.11% Coverage]

Reference 1 - 2.11% Coverage

we could all probably have a little bit more training in the various things and as far as pregnant women are concerned, people carry out with new ideas all the time and that would require more training for us to be adequately educated in those things

<Internals\\Transcript Ph#5.01> - § 2 references coded [3.59% Coverage]

Reference 1 - 0.93% Coverage

Some incentives will be good, that would help investing some time in getting more training done.

Reference 2 - 2.66% Coverage

try to invest a little bit more in creating some other options; so when pregnant or breastfeeding ladies have common problems that we can treat with over-the-counter medications…like give us options to do that, that would be great. At this stage, there aren't too many things.

<Internals\\Transcript Ph#6.01> - § 1 reference coded [1.49% Coverage]

Reference 1 - 1.49% Coverage

I guess training, and maybe having an additional module to help with pregnancy, especially as new medicines come into market, or more information comes about medications and as the categorization changes, we get more understanding and that would be beneficial

<Internals\\Transcript Ph#9.01> - § 1 reference coded [0.55% Coverage]

Reference 1 - 0.55% Coverage

it will be really great to have some sort of training on an ongoing basis, like once a year
